# Functional Analyses of a Novel Splice Variant in the *CHD7* Gene, Found by Next Generation Sequencing, Confirm Its Pathogenicity in a Spanish Patient and Diagnose Him with CHARGE Syndrome

**DOI:** 10.3389/fgene.2018.00007

**Published:** 2018-01-26

**Authors:** Olatz Villate, Nekane Ibarluzea, Eugenia Fraile-Bethencourt, Alberto Valenzuela, Eladio A. Velasco, Detelina Grozeva, F. L. Raymond, María P. Botella, María-Isabel Tejada

**Affiliations:** ^1^Biocruces Health Research Institute, Barakaldo, Spain; ^2^Molecular Genetics Laboratory, Genetics Service, Cruces University Hospital, Barakaldo, Spain; ^3^Splicing and Cancer Laboratory, Instituto de Biología y Genética Molecular, Consejo Superior de Investigaciones Científicas, Universidad de Valladolid, Valladolid, Spain; ^4^Department of Medical Genetics, Cambridge Institute for Medical Research, University of Cambridge, Cambridge, United Kingdom; ^5^Department of Pediatrics, Araba University Hospital, Vitoria, Spain; ^6^Clinical Group, Centro de Investigación Biomédica en Red de Enfermedades Raras, Madrid, Spain

**Keywords:** *CHD7*, next generation sequencing, alternative splicing, minigene, CHARGE syndrome

## Abstract

Mutations in *CHD7* have been shown to be a major cause of CHARGE syndrome, which presents many symptoms and features common to other syndromes making its diagnosis difficult. Next generation sequencing (NGS) of a panel of intellectual disability related genes was performed in an adult patient without molecular diagnosis. A splice donor variant in *CHD7* (c.5665 + 1G > T) was identified. To study its potential pathogenicity, exons and flanking intronic sequences were amplified from patient DNA and cloned into the pSAD^®^ splicing vector. HeLa cells were transfected with this construct and a wild-type minigene and functional analysis were performed. The construct with the c.5665 + 1G > T variant produced an aberrant transcript with an insert of 63 nucleotides of intron 28 creating a premature termination codon (TAG) 25 nucleotides downstream. This would lead to the insertion of 8 new amino acids and therefore a truncated 1896 amino acid protein. As a result of this, the patient was diagnosed with CHARGE syndrome. Functional analyses underline their usefulness for studying the pathogenicity of variants found by NGS and therefore its application to accurately diagnose patients.

## Introduction

The advent of next generation sequencing (NGS) technology which allows the sequencing either of the whole genome or of the expressed genes (exome) in one analysis, is transforming the process of genetic testing. NGS is being used extensively to diagnose diseases and find novel causative mutations for disease phenotypes. However, detailed analysis conclusively confirming these variants, as well as the underlying molecular mechanisms explaining the diseases, are often lacking.

Actually, 100s of 1000s of DNA variants are detected in massive sequencing projects of genetic disorders and interestingly, many estimations have shown that an unexpectedly large fraction of genetic diseases are caused by variants that disrupt the splicing process (Wang and Cooper, 2007), ranging from 15 to >60% (Lopez-Bigas et al., 2005).

*CHD7* is a gene located on chromosome 8q12.1 encoding the chromodomain helicase DNA-binding (CHD) protein 7, which belongs to a family of nine CHD proteins that can modify chromatin structure (Vissers et al., 2004). Among them CHD7 is a transcriptional regulator that binds to enhancer elements in the nucleoplasm. CHARGE syndrome is characterized by Coloboma, Heart defects, Atresia of the choanae, Retardation of growth and development, Genital hypoplasia and Ear abnormalities, and approximately 60–70% of the patients have pathogenic mutations in *CHD7*, the major causative gene of this syndrome (Zentner et al., 2010b). While most *CHD7* mutations are nonsense or frameshift and predicted to be loss of function (Zentner et al., 2010a), the incidence of splice mutations is low, around 12% according to the Human Gene Mutation Database (HGMD) (Stenson et al., 2017). Moreover, splice mutations are often based on bioinformatic predictions and functional analyses confirming the pathogenicity of the mutations are lacking. Here, we describe the functional consequences of a novel splice mutation in *CHD7* found by NGS in a patient without molecular diagnosis.

## Background

### Case Report

The male patient was the second child born to non-consanguineous Spanish healthy parents (24 and 30 years old, at the time of birth), with an unremarkable family history. He was born after 40 weeks of an uneventful pregnancy at a local hospital in 1977 and birth parameters were: weight 2,860 g (20th percentile), a length of 49 cm (5th percentile) and head circumference 36 cm (50th percentile). Apgar scores were 9 and 9 at 1 and 5 min, respectively.

At birth, he had an acceptable general appearance with good skin color, good muscle tone and normal active movements, but he showed facial dysmorphic features, including right choanal atresia resulting in a respiratory insufficiency, abnormal placement of the parietals, retromicrognathia of the lower jaw, narrow palate and glossoptosis, bilateral dysplastic, and low-set ears, protrusion of the right eye with megalocornea and papilar coloboma of the left eye. There were no thorax anomalies, neither in the limbs nor in the genitalia. At the age of 10 days, a systolic murmur was detected and therefore a congenital heart anomaly was suspected. At 21 days he was transferred to a reference hospital in Barcelona (Spain) where they found a cardiomegaly, an interventricular communication and an arteriovenous shunt and he was diagnosed with an atypical Treacher-Collins-Franceschetti syndrome.

During his 1st year of life he was admitted to his local hospital on many occasions due to breathing and swallowing difficulties requiring artificial ventilation and nasogastric tube feeding. Biochemical tests were negative, with no evidence of metabolic disease. At 1 year of age he had surgery due to his heart malformations in a reference center in Navarra (Spain).

It was early on when doctors realized that his psychomotor development was also delayed. He had autistic features and developed no speech. His weight-stature development was normal. Clinical data throughout his life are scarce but he was repeatedly admitted to the hospital because of recurrent respiratory infections, dyspnea, swallowing difficulties, gastrointestinal bleedings and a hyper-excitability which was difficult to control with a severe intellectual disability (ID). His parents had always cared for him at home until he died in 2013, at the age of 36. He had never been seen by an expert in Medical Genetics.

**FIGURE 1:**
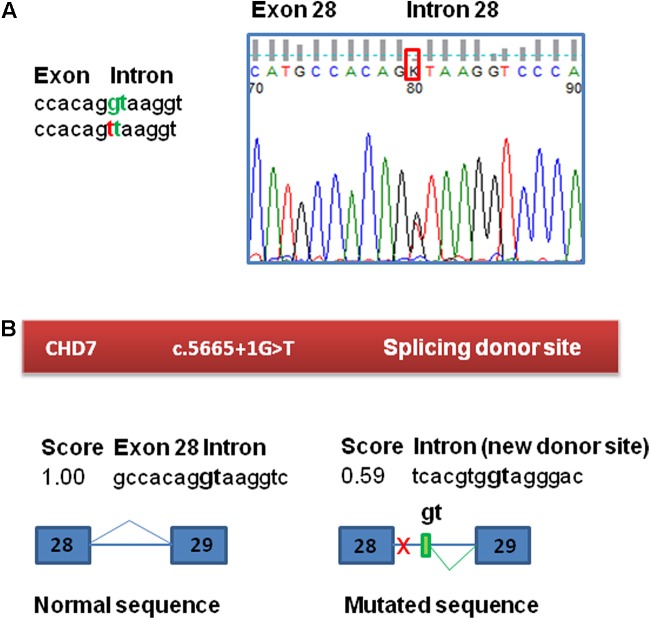
Confirmation and bioinformatic analysis of the c.5665 + 1G > T variant found in the *CHD7* gene. **(A)** Electropherogram of the genomic sequence confirming the variant identified by NGS. This variant was not found in the patient’s parents. **(B)** Bioinformatic analysis of the variant. The variant has a potential effect on the splicing process, eliminating the canonical splicing donor site of exon 28. The presence of a cryptic donor site in intron 28, 64 nt downstream (NNSplice = 0.59) is also detected.

The molecular study of this patient began in 2010 in the context of a research project. This study was approved by the ethics committee for clinical research of Araba University Hospital (Vitoria, Spain). Informed consent was obtained from his parents before the extraction of peripheral blood samples for genetic analyses and they provided written consent to publish the report. Test results for karyotype, fragile X syndrome and arrays-CGH (60k) revealed no abnormalities. In 2011, he was included in a panel sequencing study of 565 ID-related genes within the UK10K project due to the scarcity of clinical data. A novel splice mutation in the *CHD7* gene was observed: c.5665 + 1G > T (Grozeva et al., 2015). This variant was not observed in gnomAD variant frequency database of more than 100,000 sequenced individuals (Lek et al., 2016). The presence of the variant in the patient was validated by Sanger sequencing and was absent in the parents, confirming that it is a *de novo* mutation (**Figure 1A**). When the parents received the result and the altered diagnosis of CHARGE syndrome, the patient had already died. Further analysis of the variant was performed to inform the recurrence risk for extended family members.

## Materials and Methods

Functional consequences of the mutation c.5665 + 1G > T in *CHD7* were tested by a minigene assay, as RNA from the patient was not available. First, to evaluate the potential impact of the variant on splicing, Human Splicing Finder^1^ and NNSplice^2^ splicing prediction programs were used. This bioinformatic analysis of the c.5665 + 1G > T variant in *CHD7* predicted the disruption of the canonical splice donor of exon 28 and also detected the presence of a cryptic donor site in the intron 28, 64 nucleotides downstream (NNSplice = 0.59) (**Figure 1B**). The +1G nucleotide is conserved in 100% of the 5′splice site recognized by the major spliceosome.

**FIGURE 2:**
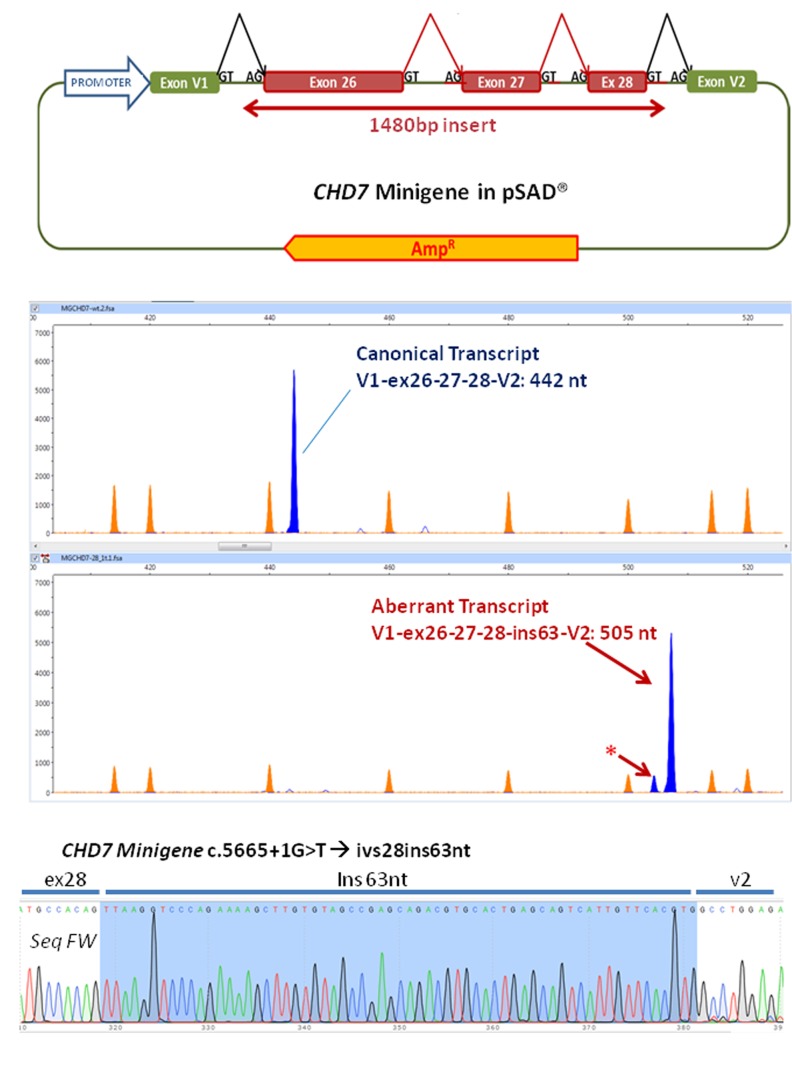
Functional analysis of the variant using a minigene strategy. **(A)** Exons 26–28, complete 26 and 27 introns and part of flanking introns 25 and 28 (intron 25, 355 bp, intron 28, 298 bp) of the *CHD7* gene were amplified (total size 1480 bp) and cloned into the splicing vector pSAD^®^. V1 and V2 are constitutive exons of the vector that in eukaryotic cells produce the splicing reactions (marked in the figure) with the cloned exons. **(B)** The RT-PCR functional assay of the wild-type (above) and mutant (below) minigenes was performed with a FAM-labeled first fluorescence (blue peaks). Capillary electrophoresis was performed in triplicate on an automated sequencer with the Genescan LIZ 1200 size standard (orange peaks; Applied Biosystems) and Peak Scanner (Applied Biosystems) analysis. The asterisk indicates a possible aberrant transcript. **(C)** Partial sequence of the RT-PCR product generated by the c.5665 + 1G > T variant. After sequencing, the insertion of 63 nt of intron 28 can be observed. No traces of other aberrant transcripts detected in the fragment analysis were observed. In the upper right the sequence of the border between exons 28 and V2 of the canonical transcript produced by the wild-type minigene can be observed.

A minigene was constructed with an insert of 1480 bp corresponding to the exons 26, 27 and 28 and the flanking intronic sequences of the *CHD7* gene with the following structure: ivs25 (355 bp)-ex26 (130 bp)-ivs26 (409 bp)-ex27 (73 bp)-ivs27 (157 bp)-ex28 (58 bp)-ivs28 (298 bp) (**Figure 2A** and Supplementary Figure S1). Briefly this insert was amplified with the primers FW: 5′ GGTGGCGGCCGCTCTAGAACTAGTGGATCCCCCGG GCAGAGGTCATAAAGGAACATT 3′ and RV: 5′ GACGGTATCGATAAGCTTGATATCGAATTCCTGCACAAATGCTCTATGCTCTATTCCC 3′ (cloning tails are underlined) and the high fidelity polymerase (Phusion Hot Start) from the patient’s DNA.

Fragments were cloned into the splicing vector pSAD^®^ (Acedo et al., 2015) between the EagI y ClaI sites (minigene MGchd7_ex26–28) and the complete insert was sequenced to check the presence of the wild-type allele or the mutant one. Using a standard protocol of transfection, approximately 10^5^ HeLa cells were transfected with the wild-type and the mutant minigenes. To inhibit nonsense-mediated decay (NMD), cells were incubated with cycloheximide (CHX). RNA was extracted after 48 h and purified with the Genematrix Universal RNA purification Kit (EURx, Gdansk, Poland) with on-column DNAse I digestion to degrade genomic DNA that could interfere in RT-PCR. Retrotranscription was carried out with specific primers of exons V1 and V2 of the pSAD^®^ vector as described (Acedo et al., 2015; Fraile-Bethencourt et al., 2017). Samples were sequenced at the Macrogen facility (Macrogen Spain, Madrid, Spain). Fragment analysis was carried out with Peak Scanner v1.0 (Life Technologies). Mean peak areas of each transcript and standard deviations were calculated.

## Functional Analysis Results

Functional analysis of the wild-type minigene (MGchd7_EX26-28) revealed the expected canonical transcript [(442 nt = exons V1 (84 nt)- ex 26 (130 nt)- ex27 (73 nt)- ex28 (58 nt)- V2 (97 nt)] while the construct with the variant c.5665 + 1G > T produced a principal aberrant transcript (**Figure 2B**). The sequence of the RT-PCR product generated from the mutant minigene showed the insertion of 63 nucleotides of intron 28 by use of a 64 nt downstream alternative donor site (r.5665_5666ins5665 + 1_5665 + 63) (**Figure 2C**). The effect on the protein would be the insertion of eight new amino acids (VKVPEKLV) after the position Thr1888 and the appearance of a pre-termination codon (TAG) 25 nucleotides downstream (p.Gly1889ValfsTer8), resulting in a truncated 1896 amino acid protein.

## Discussion

Next generation sequencing-based target sequencing has the potential to serve as a powerful tool that allows definitive diagnosis. Despite numerous studies, there is still a huge challenge in deciding whether or not variants detected by NGS are pathogenic. Although the rapidly evolving bioinformatic methods help in the identification of potential functional variants from large data sets, functional analyses to test these predictions are essential. Here, we present a case of a patient without molecular diagnosis, but with a clinical diagnosis of atypical Treacher-Collins-Franceschetti syndrome. The patient was included in a screening of 986 individuals with moderate to severe ID for variants in 565 known or candidate ID-associated genes using targeted NGS within the UK10K project (Grozeva et al., 2015). A novel splice site mutation in *CHD7* was found in this patient reclassifying him as having CHARGE syndrome. This was very important because CHARGE syndrome was first described years after the patient’s birth and therefore his clinicians did not know of this syndrome at that time. So even if the patient presented with three out of four major clinical signs for this syndrome at birth, he was not diagnosed until the NGS study. Moreover, an overlap has been described of clinical features with many other diseases, such as 22q deletion syndrome, Kabuki syndrome, Kallmann syndrome, retinoic acid embryopathy, VACTERL association and PAX2 abnormalities (Lalani et al., 1993; Kohmoto et al., 2016). In patients who do not completely fulfill the clinical CHARGE diagnostic criteria, the identification of *CHD7* mutations is important in order to guarantee accurate clinical surveillance, which can possibly lead to the description of additional CHARGE features (Janssen et al., 2012).

Previous studies have reported that mutations in *CHD7* are the major cause of CHARGE syndrome (Vissers et al., 2004; Zentner et al., 2010b; Lee et al., 2016). *CHD7* mutations in typical CHARGE syndrome patients occur *de novo* in the vast majority of the cases (Lalani et al., 1993). Haploinsufficiency for CHD7 is the most likely pathogenic mechanism of this syndrome (Kohmoto et al., 2016). In HGMD Professional 2017.2, 757 *CHD7* mutations have been reported in CHARGE syndrome, 96 of them being splice mutations (Stenson et al., 2017). The c.5665 + 1G > T variant is not reported in HGMD and our patient is the first described with this variant although there are several splice variants in that region.

To confirm the pathogenicity of the novel mutation, functional assays were performed. The feasibility of performing functional analyses depends on the availability and accessibility of the required samples which can be a major challenge. In this context, a large number of methods, including model systems, can be used for functional interpretation of genome sequence variants. In our case, bioinformatic analyses suggested that the mutation could affect the splicing process. The ideal manner to study it would be to use the patient’s RNA but in this case, it was impossible to obtain because the patient was already deceased. Subsequently, a reliable and straightforward method to assess splicing was required. *Ex vivo* assays of DNA variants with splicing reporter minigenes have emerged to solve this problem (Bottillo et al., 2007; Lu et al., 2007; Jaijo et al., 2011; Acedo et al., 2015). Minigenes allow precise quantification of a single-mutant allele effect without the interference of the wild-type counterpart in patient samples. Another advantage of this approach is the high reproducibility of physiological/pathological splicing patterns by virtue of keeping the genomic context of each exon. Our functional assay showed that the novel splice donor variant c.5665 + 1G > T has a complete impact on the splicing of the *CHD7* gene and the effect would be a truncated protein.

The large 2997 amino acid CHD7 protein contains two chromodomains at its N terminus, followed by centrally located SNF2 and helicase domains; three conserved region (CR) domains; a switching-defective protein 3, adaptor 2, nuclear receptor corepressor, transcription factor IIIB (SANT) domain; two Brahma and Kismet (BRK) domains of unknown function; and, at the C terminus a leucine-zipper domain (Kim et al., 2008). It has been previously described that CHD7 can bind to the p53 promoter, thereby negatively regulating p53 expression, and that CHD7 loss in mouse neural crest cells or samples from patients with CHARGE syndrome results in p53 activation (Van Nostrand et al., 2014). The effect of the splice mutation found in this patient on the CHD7 protein will be the loss of the SANT and the BRK domains. The SANT domain may mediate binding to either DNA or modified histones (Schnetz et al., 2009) so the truncated protein will lose the ability to bind to the DNA or histones.

The minigene-construct allows the analysis of multiple variants from different exons. Therefore, the minigene containing exons 26–28 and the flanking intronic sequences of the *CHD7* gene we have constructed, could be used for the analysis of other splice mutations in that region for which there are not functional analysis yet (Bartels et al., 2010).

## Concluding Remarks

Functional analyses are very useful for studying the pathogenicity of variants found by NGS. In the case of novel mutations in the *CHD7* gene, the analysis of the functional consequences is very useful not only in subjects with typical signs not recognized at birth like our case, but also in subjects with atypical clinical signs. Specifically, in the present study our findings demonstrate that the novel variant c.5665 + 1G > T has a complete impact on the splicing of the *CHD7* gene resulting in a CHARGE syndrome and therefore improves our understanding of the genetic causes of CHARGE syndrome which is useful for accurately diagnosing patients and for providing genetic counseling to families.

## Author Contributions

MB provided patient clinical data and samples; EF-B, AV, EV, DG, FR, OV, and NI collected data and performed the experiments; OV and M-IT designed the study. All authors revised the manuscript critically, approved the final manuscript as submitted and agreed to be accountable for all aspects of the work.

## Conflict of Interest Statement

The authors declare that the research was conducted in the absence of any commercial or financial relationships that could be construed as a potential conflict of interest.

## Footnotes

1 http://www.umd.be/HSF/

2 http://www.fruitfly.org/seq_tools/splice.html
