# Supplementary figures and images for: Functional Analyses of a Novel Splice Variant in the CHD7 Gene, Found by Next Generation Sequencing, Confirm Its Pathogenicity in a Spanish Patient and Diagnose Him with CHARGE Syndrome
Source: Front Genet. 2018 Jan 26;9:7. doi: 10.3389/fgene.2018.00007 (PMC5790995; doi:10.3389/fgene.2018.00007)

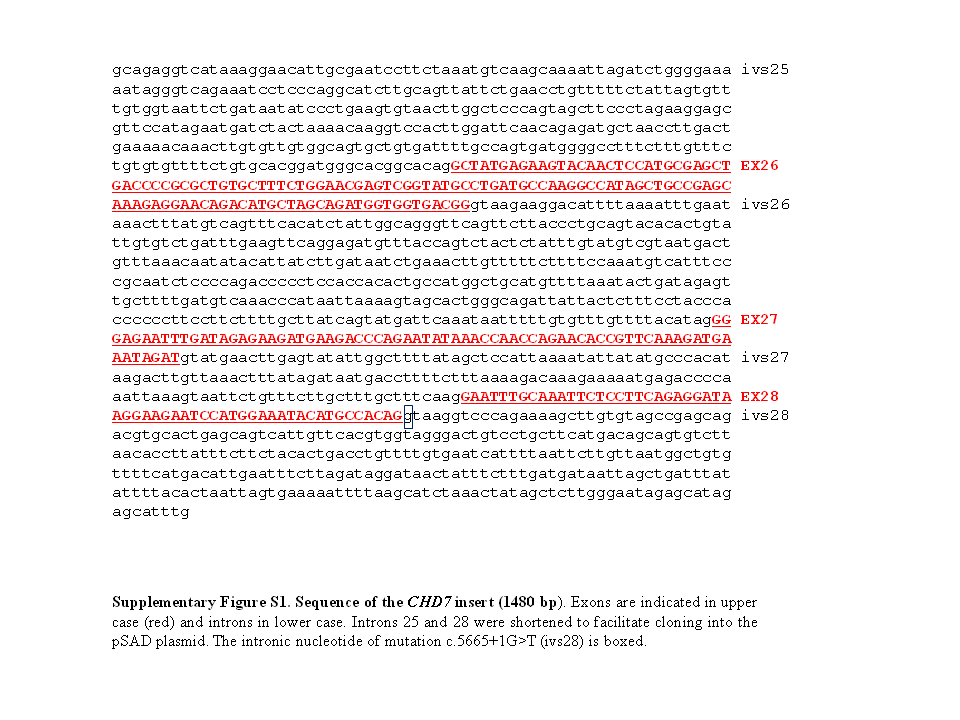

Supplement: Supplementary file 1 [file Image_1.TIF]
